# Fosfomycin enhances phagocyte-mediated killing of *Staphylococcus aureus* by extracellular traps and reactive oxygen species

**DOI:** 10.1038/srep19262

**Published:** 2016-01-18

**Authors:** Fengge Shen, Xudong Tang, Wei Cheng, Yang Wang, Chao Wang, Xiaochen Shi, Yanan An, Qiaoli Zhang, Mingyuan Liu, Bo Liu, Lu Yu

**Affiliations:** 1 Key Laboratory of Zoonosis Research, Ministry of Education, Institute of Zoonosis, The First Hospital of Jilin University, College of Veterinary Medicine and College of Animal Science, Jilin University, Changchun, China; 2 Key Lab for New Drug Research of TCM, Research Institute of Tsinghua University in Shenzhen, Shenzhen, China; 3 Department of Infectious Diseases, First Hospital of Jilin University, Changchun, China; 4 Jiangsu Co-innovation Center for Prevention and Control of Important Animal Infectious Diseases and Zoonoses, Yangzhou, China

## Abstract

The successful treatment of bacterial infections is the achievement of a synergy between the host’s immune defences and antibiotics. Here, we examined whether fosfomycin (FOM) could improve the bactericidal effect of phagocytes, and investigated the potential mechanisms. FOM enhanced the phagocytosis and extra- or intracellular killing of *S. aureus* by phagocytes. And FOM enhanced the extracellular killing of *S. aureus* in macrophage (MФ) and in neutrophils mediated by extracellular traps (ETs). ET production was related to NADPH oxidase-dependent reactive oxygen species (ROS). Additionally, FOM increased the intracellular killing of *S. aureus* in phagocytes, which was mediated by ROS through the oxidative burst process. Our results also showed that FOM alone induced *S. aureus* producing hydroxyl radicals in order to kill the bacterial cells *in vitro*. In a mouse peritonitis model, FOM treatment increased the bactericidal extra- and intracellular activity *in vivo*, and FOM strengthened ROS and ET production from peritoneal lavage fluid *ex vivo*. An IVIS imaging system assay further verified the observed *in vivo* bactericidal effect of the FOM treatment. This work may provide a deeper understanding of the role of the host’s immune defences and antibiotic interactions in microbial infections.

*Staphylococcus aureus* (*S. aureus*) is a gram-positive extracellular and facultative intracellular bacterium that causes a large number of difficult-to-treat nosocomial infections^1^. Many of these infections, especially those related to implanted medical devices, are often linked to the formation of biofilms and are accompanied by chronic or recurrent characteristics^2^. Biofilms are complex bacterial communities embedded in an extracellular matrix that allows bacteria to resist host responses and antimicrobial agents^3^,^4^. Based on its pathogenicity and epidemicity, *S. aureus* has increasingly been used as a bacterial model in studies that aim to investigate antibiotic or innate immune mechanisms^5^,^6^.

The successful treatment of bacterial infections is the achievement of a synergy between the host’s immune defences and antibiotics, so anti-infective regimens must consider the combination of the host’s immune response with antimicrobial drugs in order to be effective^7^. Fosfomycin (FOM), a broad-spectrum antibacterial agent possessing a unique chemical structure, unique pharmacologic features and a lack of toxicity, is a promising drug for clinical use. FOM has been shown to be a bactericidal drug^8^ and to have activity against methicillin-resistant *S. aureus* strains *in vitro* and *in vivo*^9^,^10^,^11^. FOM also significantly affected *S. aureus* biofilm cell viability^12^. Previous studies demonstrated that a synergistic interaction against *S. aureus* or *Streptococcus pneumoniae* exists between human phagocytes with sub-inhibitory concentrations of some antibiotic agents, such as penicillin, clindamycin, or cephalosporins^13^,^14^. Based on these findings, we found that FOM significantly enhanced the bactericidal activity of macrophages (MФ) and human neutrophils against *S. aureus*; however, the concrete molecular mechanisms of the synergistic killing bacteria between FOM and phagocytes have not been reported.

Innate immunity is an important part of host defence in the elimination of infections that are caused by pathogens. Neutrophils and MФ are professional phagocytes^15^. Neutrophils and MФ engulf microbes at the site of infection into phagosomes. Then, phagolysosomes are generated, in which the pathogens are killed after exposure to lytic enzymes, antimicrobial peptides and reactive oxygen species (ROS)^16^. ROS are produced by a process called oxidative burst, which is mediated by the NADPH oxidase2 (NOX2) complex^17^. ROS includes the superoxide anion, hydrogen peroxide and hydroxy radicals^18^. Moreover, bactericidal antibiotics (e.g., ampicillin, kanamycin, and norfloxacin) were reported to induce highly deleterious hydroxyl radical formation in bacteria, which ultimately contributed to bacteria cell death^19^,^20^.

In addition to active phagocytosis and intracellular killing by ROS, extracellular trap (ET) formation by neutrophils and MФ cells, which is a novel cell death pathway called ETosis, provides an extracellular site for microbial killing in the innate immune defense^21^. After stimulation, the chromatin from these cells undergo decondensation followed by mixing of euchromatin and heterochromatin, then eject their own chromatin content mixed with their granular components in the extracellular space to form meshwork of extracellular DNA (eDNA) fibers (extracellular reticulate structures called extracellular traps; ETs) that are covered with antimicrobial molecules, including elastase and myeloperoxidase, which capture and kill bacteria, fungi, and parasites^22^. ET formation requires the production of ROS^22^. Human and murine neutrophils and monocytes/MФ can kill *S. aureus* through the ET production, and some drugs, such as statin, boost this phenotype^23^.

To address whether the interactions between FOM and phagocytes against *S. aureus* are related to the aspects mentioned above, we determined the characteristics and possible mechanisms of their synergistic effects *in vitro* and *in vivo*, including ET formation and ROS production.

## Results

### Phagocyte and FOM bactericidal effects against *S. aureus* biofilms and PLK cells

The susceptibility assay demonstrated that FOM had antibacterial activities against planktonic (PLK), resuspended biofilm (RBF), and biofilm cell cultures from 4 representative *S. aureus* strains RN6390, Xen29, SA113, and SA113∆*ica* (biofilm deficient) (Table 1). The minimum inhibitory concentration (MIC) values for the FOM treatment against PLK, RBF, biofilm cells of the 4 strains were 16 μg/ml, 16 ~ 32 μg/ml, and 32 ~ 256 μg/ml, respectively. Additionally, the minimum bactericidal concentration (MBC) values for the FOM treatment against the PLK, RBF, and biofilm cells of the 4 strains were 32 ~ 256 μg/ml, 128 ~ 512 μg/ml, and > 1024 μg/ml, respectively. These data showed that the anti-staphylococcal activity *in vitro* of FOM against biofilm cells were weaker than that of planktonic cells.

Additionally, the total damage percentage that was caused by phagocytes to *S. aureus*, RBF, or PLK cells were detected with an XTT (2,3-bis[2-methoxy-4-nitro-5-sulfophenyl]2H-tetrazolium-5-carboxanilide) assay at 37 °C for 22 h (Table 2). The damage to the RBF cells that was caused by mouse MФ and human neutrophils were significantly lower compared with that of the PLK cells (when the effector to target cell (E:T) ratios was 10:1, 1:1, and 1:5) (*p* < 0.05, Table 2). However, when the E:T ratio was 1:10, both the MФ and human neutrophil-induced RBF cell damage was not significantly different compared with PLK cell induced damage (Table 2). These results showed that RBF cells have a strong anti-phagocytic ability against phagocytes compared with PLK cells, and further, that this ability was related to the E:T ratio.

**Figure 1:**
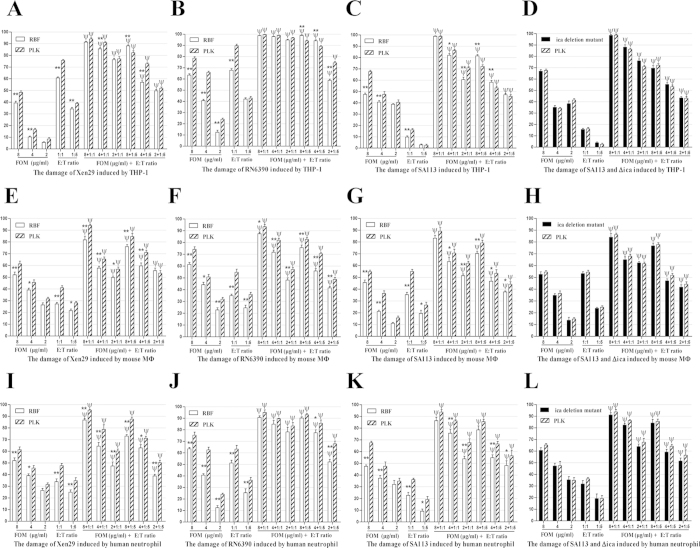
FOM enhanced the phagocyte bactericidal effect against *S. aureus* biofilms and PLK cells. The damage induced by human THP-1 cells or with FOM against Xen29 (**A**), RN6390 (**B**), SA113 (**C**) (striped bar) or SA113 Δ*ica* (**D**, black bars) RBF (white bars) or PLK cells, mouse MФ or with FOM against Xen29 (**E**), RN6390 (**F**), SA113 (**G**) or SA113Δ*ica* (**H**) RBF or PLK cells, human neutrophils or with FOM against Xen29 (**I**), RN6390 (**J**), SA113 (**K**) or SA113 Δ*ica* (**L**) RBF or PLK cells after incubation at 37 °C for 22 h at different effector to target cell (E:T) ratios. The data are means ± standard errors (error bars), which were derived from three experiments. The result of the damage induced by the combination of MФ and FOM was compared with the human THP-1 cells or MФ alone results by analysis of variance with the One-Way ANOVA test. ^ψ^*p* < 0.05 represents the significant difference between FOM (2 μg/ml, 4 μg/ml, 8 μg/ml) + RBF/PLK and RBF/PLK/FOM alone conditions. *p < 0.05 represents the significant difference between RBF (or isogenic *ica* deletion mutant) and PLK conditions. **p < 0.01 represents the significant difference between RBF (or isogenic *ica* deletion mutant) and PLK conditions

Further, the FOM concentration dependently enhanced (2 μg/ml to 8 μg/ml) the total *S. aureus* RBF or PLK cell damage by MФ or human neutrophils (*p* < 0.05, Fig. 1). FOM alone at various concentrations (2 μg/ml, 4 μg/ml and 8 μg/ml) inhibited the growth of the 4 tested RBF or PLK cell strains. The damage to the RBF cells that was induced by FOM alone at 8 μg/ml (1/2 MIC) or 4 μg/ml (1/4 MIC) was significantly lower than that to the PLK cells in these strains (*p* < 0.05). In the SA113 and Xen29 strains, there were no significant FOM induced total damage differences between the RBF and PLK cells at 2 μg/ml (1/8 MIC). However, there were also no significant total damage differences between the SA113 and SA113∆*ica* (a biofilm deletion strain) cells that were induced by MФ or human neutrophils alone or in combination with FOM (Fig. 1D,H,L). Together, all of these results suggested that FOM increased the bactericidal effect of MФ or human neutrophils against *S. aureus* RBF or PLK cells.

### FOM strengthened the NADPH oxidase-dependent ET production and extracellular killing of *S. aureus* by ETs

**Figure 2:**
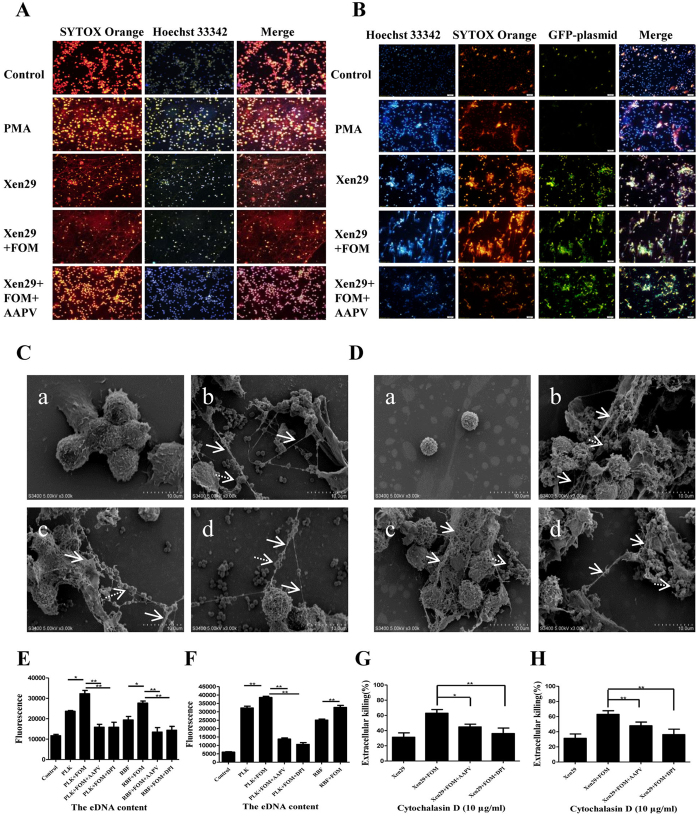
FOM strengthened the NADPH oxidase-dependent ET production and extracellular killing of *S. aureus* by ETs. (**A,B**) Fluorescence microscopy images of ET production from mouse peritoneal MФ (**A**) or human neutrophil (**B**) in the presence of the elastase inhibitor, AAPV, were visualized with fluorescence microscopy with a 20× objective lens. ET formation was visualized in red (SYTOX Orange), *S. aureus* Xen 29 with a GFP–producing plasmid was visualized in green and mouse peritoneal MФ was visualized in blue with the Hoechst 33342 dye. (**C,D**) SEM micrographs of human THP-1 cells (**C**) and neutrophils (**D**) interacting with *S. aureus* alone (b) or FOM (c) at 37 °C for 3 h, or in the presence of the elastase inhibitor, AAPV (d), were visualized by scanning electron microscopy with at a magnification of 3000x. Human THP-1 cells (a) and neutrophils (a) alone were used as control group, respectively. The solid and dashed arrow indicated ETs and *S. aureus*, respectively. (**E**) The eDNA content from mouse peritoneal MФ was quantified and stimulated with *S. aureus* PLK and RBF cells alone or with FOM, or in the presence of the elastase inhibitor, AAPV, or the NADPH oxidase inhibitor, DPI. (**F**) The eDNA content was quantified from human neutrophils that were stimulated with *S. aureus* PLK and RBF cells alone or with FOM, or in the presence of the elastase inhibitor, AAPV, or the NADPH oxidase inhibitor, DPI. (**G**,**H**) The percentage of extracellular *S. aureus* killing by ETs from mouse peritoneal MФ (**G**) or human neutrophils (**H**) was analysed, and they included Xen29 or FOM (4 μg/ml)-treated Xen29 groups in the presence of AAPV or DPI, and they were treated with actin inhibitor Cytochalasin D (10 μg/ml) to block phagocytosis. The data are means ± standard errors derived from three experiments. Comparisons between groups were performed using the One-Way ANOVA test. **p < 0.01, *p < 0.05.

As mentioned above, ET formation from neutrophils and MФ provides an extracellular site for microbial killing as an innate immune defense^21^. Phagocytes can kill extracellular bacteria by ET release, which traps bacteria covered with antimicrobial molecules, including elastase and myeloperoxidase. To analyse whether FOM enhanced the ETs production in *S. aureus*-infected phagocytes, representative fluorescent images of phagocytes that were treated with GFP-expressing Xen29 cells alone or co-incubated with FOM for 3 h at 37 °C were collected. MФ without bacteria did not produce METs, and PMA did produce METs (positive control), whereas Xen29 induced MФ to produce reticulate structures, with some *S. aureus* being trapped by METs (Fig. 2A). With the FOM treatment, the Xen29-infected MФ produced more intense reticulate structures (Fig. 2A). FOM also enhanced the NETs production from human neutrophils following *S. aureus* infection (Fig. 2B). Additionally, fluorescence analysis demonstrated that the elastase inhibitor, AAPV (methoxysuccinyl-Ala-Ala-Pro-Val-chloromethyl ketone; Sigma, St. Louis, MO, USA), significantly reduced ETs production that was induced by FOM in the *S. aureus*-infected mouse peritoneal MФ or human neutrophils (Fig. 2A,B). Further, scanning electron microscope (SEM) micrographs also verified a few ETs were produced by MФ or human neutrophils following *S. aureus* infection, and FOM treatment induced more ETs bacteria entrapment by the *S. aureus*-infected MФ or human neutrophils. AAPV significantly inhibited the phagocyte ET production (Fig. 2C,D).

To quantify the MФ extracellular traps (MET) formation, we analysed the extracellular DNA (eDNA) content in the supernatants of *S. aureus* cells and MФ co-cultures, and in the presence of FOM (Fig. 2E). The MФ that were stimulated by PLK/RBF cells alone or with FOM produced 2.0, 2.7, 1.7, and 2.4 fold more eDNA content than that from untreated MФ, respectively (Fig. 2E). Moreover, the eDNA content that was produced from *S. aureus*-infected human neutrophils were also significantly increased with FOM treatment (Fig. 2F). The eDNA content produced from *S. aureus*-infected mouse peritoneal MФ (Fig. 2E) or human neutrophils (Fig. 2F) were significantly decreased by the elastase inhibitor, AAPV, in the presence of FOM. This result indicated that FOM could enhance the *S. aureus*-infected MФ or human neutrophil ET production.

Additionally, a phagocytosis inhibition assay was conducted to assay the effect of FOM treatment on extracellular *S. aureus* killing by ETs. The mean extracellular *S. aureus* killing by MФ was increased with FOM treatment from 31.3% to 62.9%, and it dropped to 44.9% after 3 h of AAPV treatment (Fig. 2G). Additionally, the mean extracellular *S. aureus* killing by human neutrophils was also markedly increased with the FOM treatment (Fig. 2H). These results demonstrated that FOM could strengthen extracellular *S. aureus* killing by ETs.

To investigate the possible mechanism of the phagocyte ETs formation that was induced by FOM, diphenylene iodonium (DPI; Sigma) DPI was used as an NOX2 inhibitor to assay the relationship between NADPH oxidase and ETs. We found that the addition of DPI (10 μM) almost abrogated ET formation in the FOM-treated or in FOM/*S. aureus*-treated MФ or human neutrophils (*p* < 0.01, Fig. 2E,F). At the same time, the mean extracellular *S. aureus* killing from MФ and human neutrophils dropped to 36.1% and 27.5% after 3 h of DPI treatment, respectively (Fig. 2G,H). This suggested that ET production from *S. aureus*-treated phagocytes with FOM treatment was dependent on NADPH oxidase.

### FOM enhanced phagocytosis and oxidative burst-mediated intracellular killing of *S. aureus* by phagocytes

**Figure 3:**
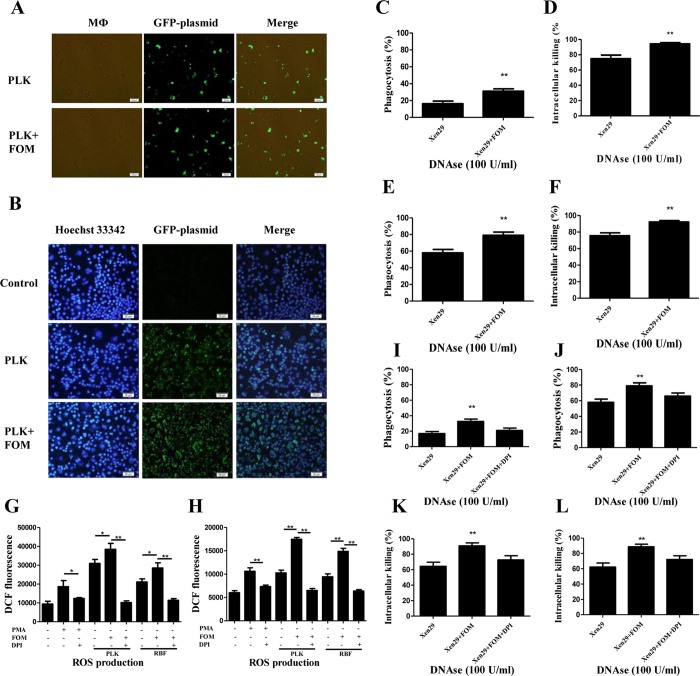
FOM enhanced phagocytosis and oxidative burst-mediated intracellular killing of *S. aureus* by phagocytes. (**A**) A phagocytosis assay was visualized by fluorescence microscopy in GFP-tagged *S. aureus* Xen29-infected murine MФ following FOM treatment at 37 °C for 2 h with a 40× objective lens. (**B**) The phagocytosis assay was visualized with fluorescence microscopy in GFP-tagged *S. aureus* PLK-infected murine MФ following FOM treatment at 37 °C for 6 h with a 20× objective lens. MФ were stained with Hoechst 33342. (**C,E**) The murine MФ (**C**) or human neutrophils (**E**) phagocytosis percentages were analysed, and they included Xen29 or FOM (4 μg/ml)-treated Xen29 groups that were treated with 100 U/ml DNase. (**D,F**) The murine MФ (**D**) or human neutrophils (**F**) intracellular killing percentages were analysed, and they included Xen29 or FOM (4 μg/ml)-treated Xen29 groups that were treated with 100 U/ml DNase. Differences of phagocytosis, intracellular killing percentages induced by murine MФ or human neutrophils between Xen29 and FOM-treated Xen29 conditions were analysed by the Student *t* test (**C,D,E** and **F**). Differences with 2-sided were considered statistically significant. (**G,H**) ROS production was quantified from THP-1 cells (**G**) or human neutrophils (**H**) in combination with FOM (4 μg/ml) against *S. aureus* RBF or PLK cells, or in the presence of DPI at 37 °C for 3 h. PMA was used as positive group. (**I,J**) The MФ (**I**) or human neutrophils (**J**) phagocytosis percentages were analysed, and they included Xen29 or FOM (4 μg/ml)-treated Xen29 groups that were treated with 100 U/ml DNase in the presence of DPI. (**K,L**) The by THP-1 cell (**K**) or human neutrophil (**L**) intracellular killing percentages were treated with 100 U/ml DNase and analysed in the presence of DPI. The data are presented as the means ± standard errors of three independent experiments by One-Way ANOVAs. **p < 0.01 compared between interest groups. *p < 0.05 compared between interest groups.

In addition to extracellular killing by ETs, phagocytosis by phagocytes is a highly effective host defence mechanism for *S. aureus* clearance^17^. In this study, after co-incubation with MФ and *S. aureus* at 37 °C for 2 h, light microscopy image analysis showed that a few *S. aureus* were devoured by murine MФ. However, with FOM treatment, a large number of *S. aureus* were phagocytized by MФ (Fig. 3A). Further, after 6 h, the images observed by fluorescence microscopy showed that a large number of *S. aureus* were phagocytized by murine MФ after FOM treatment compared with the non-FOM treated group (Fig. 3B). This result showed that FOM could increase the phagocytic ability of MФ.

The efficient uptake of bacteria by phagocytes is often followed by bactericidal action. To further corroborate whether FOM affected the intracellular killing of *S. aureus* by murine MФ, DNase I was used to eliminate extracellular killing bacteria by MET formation. The results showed that FOM treatment significantly increased the percentage of *S. aureus* phagocytosis in the presence of DNase I (*p* < 0.01, Fig. 3C). Furthermore, the mean intracellular *S. aureus* killing by DNase-treated murine MФ increased from 74.8% to 95.8% after 3 h of FOM treatment (*p* < 0.01, Fig. 3D). Additionally, the phagocytosis percentage and mean intracellular killing by DNase-treated human neutrophils were markedly increased following FOM treatment (Fig. 3E,F). This result further verified that FOM could increase the phagocytosis and intracellular killing of *S. aureus* by phagocytes.

Previous reports demonstrated that a major intracellular bactericidal mechanism used by phagocytes is the generation of ROS via oxidative burst^17^,^24^,^25^,^26^,^27^,^28^. Oxidative burst analysis showed that FOM induced much more ROS in *S. aureus* RBF or PLK cell-treated THP-1 cells or neutrophils than these phagocytes alone (*p* < 0.05, Fig. 3G,H), whereas the NADPH oxidase inhibitor DPI (10 μM) significantly inhibited these ROS production levels (*p* < 0.05, Fig. 3G,H). Additionally DPI treatment also significantly reduced the enhanced phagocytosis by phagocytes (*p* < 0.05, 3I, J) and intracellular killing by phagocytes following FOM treatment (*p* < 0.05, 3K, L). This result suggested that the enhancement of phagocytosis and intracellular killing by phagocytes with FOM was related to ROS production via the oxidative burst process.

### FOM induced hydroxyl radical production in *S. aureus*

**Figure 4:**
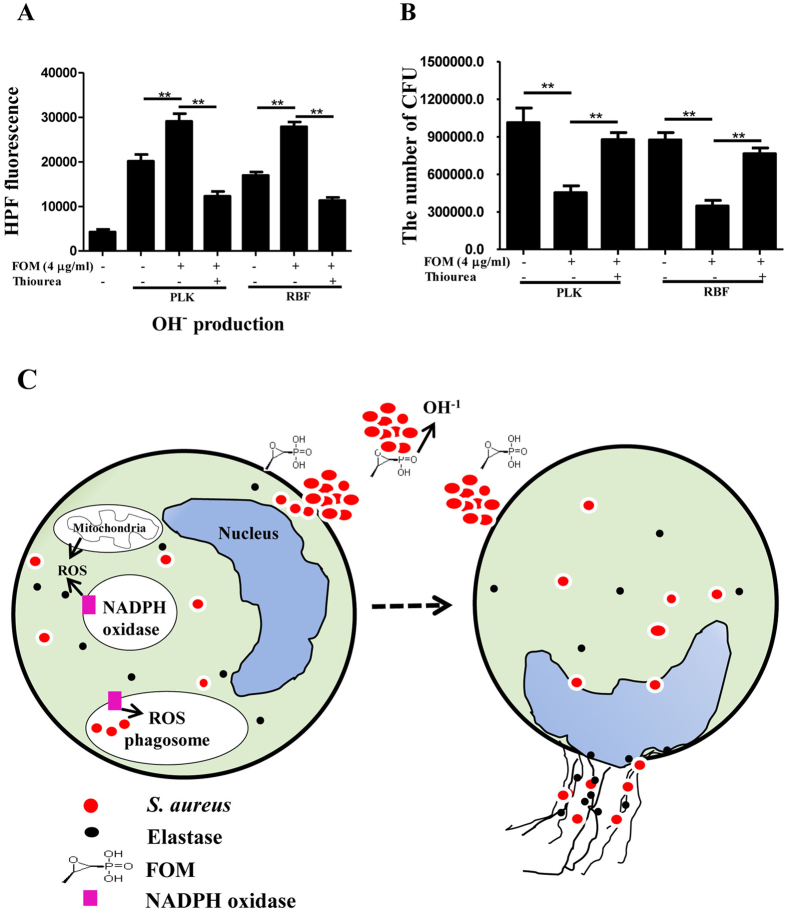
FOM caused hydroxyl radical production in *S. aureus* cells. (**A**) Hydroxyl radical (OH^-^) production was quantified with HPF in *S. aureus* PLK and RBF cells alone or with FOM treatment, or in the presence of the hydroxyl radical scavenger, thiourea. The fluorescence intensity of a hydroxyl radical probe (HPF) was measured with a fluorescence plate reader. (**B**) The *S. aureus* CFU numbers were detected with TSB agar plates following FOM treatment in the presence of the hydroxyl radical scavenger, thiourea. (**C**) The possible mechanisms included FOM enhanced phagocyte-mediated extra- and intracellular killing of *S. aureus* or that FOM directly acted on the bacteria cells. The dashed arrow indicates whether the cells process ends in ETosis, was determined by the level of intracellular ROS following FOM treatment. The data are presented as the means ± standard errors of three independent experiments. **p < 0.01 compared with interest group and interest group by One-Way ANOVA tests.

Recently, a new mechanism was reported that bactericidal antibiotic-induced stresses caused the production of highly deleterious hydroxyl radicals, which contributed to bacterial killing^19^,^29^. Based on this, we evaluated the effect of FOM, as it is a bactericidal agent, on hydroxyl radical production and bactericidal activity against *S. aureus* (Fig. 4). The results showed that *S. aureus* RBF or PLK cells alone induced hydroxyl radical production (*p* < 0.01), and the addition of FOM significantly enhanced the hydroxyl radical production (*p* < 0.01, Fig. 4A) and significantly decreased the *S. aureus* CFUs. However, the hydroxyl radical scavenger, thiourea (100 mM; Sigma, St. Louis, MO, USA), markedly inhibited the hydroxyl radical production that was induced by the combination of RBF/FOM or PLK/FOM (*p* < 0.01, Fig. 4A), and thiourea markedly protected the FOM-treated *S. aureus* killing level (*p* < 0.01, Fig. 4B). These results showed that FOM might enhance *S. aureus* killing by hydroxyl radical production from *S. aureus* cells.

Overall, considering the former results, we proposed a schematic regarding the possible mechanisms of FOM enhanced phagocyte-mediated extracellular and intracellular *S. aureus* killing or direct FOM-mediated actions on bacteria cells (Fig. 4C). Against extracellular *S. aureus*, FOM enhanced the phagocyte killing capacity through the release of eDNA, which was covered with granule contents, such as elastase. This ET release and formation required NADPH oxidase-dependent ROS. Against intracellular *S. aureus*, FOM enhanced the *S. aureus* engulfment capacity by phagocytes and promoted a strong oxidative burst, which was initiated by NADPH-dependent oxidases in the phagocytes, leading to the generation of highly toxic ROS, which plays an important role in the intracellular killing of *S. aureus*. In other words, FOM increased the bactericidal effect through ROS and ETosis in *S. aureus* infected-phagocytes. Importantly, whether or not the cell process ends in ETosis was determined by evaluating the intracellular ROS level following FOM treatment^30^. However, FOM itself also enhanced the *S. aureus* killing that was induced by hydroxyl radical production in the bacterial cells.

### FOM enhanced extra- and intracellular bacterial killing in a mouse peritonitis model *in vivo*

**Figure 5:**
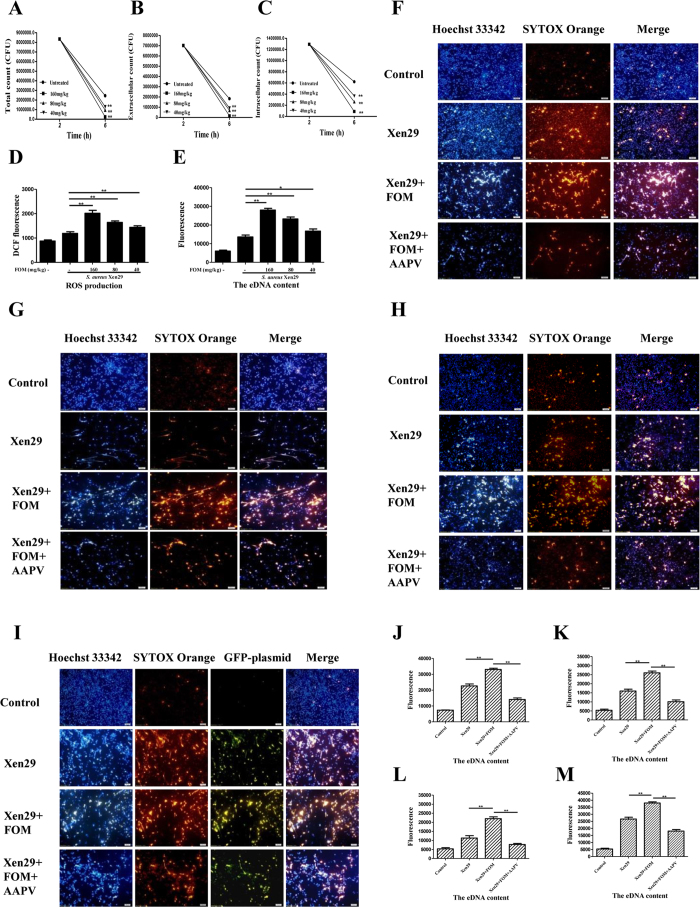
FOM enhanced extra- and intracellular bacterial killing in a mouse peritonitis model *in vivo.* (**A–C**) Total (**A**), extra- (**B**) and intracellular (**C**) time-kill studies evaluating FOM against *S. aureus* were performed in the mouse peritoneum. Mice were inoculated i.p. with *S. aureus,* Xen29. The mice were then treated s.c. with a different FOM dose. (**D**) ROS production quantification within the peritoneal lavage fluid was detected with 5 μM DCFH-DA on a fluorescence plate reader. (**E**) The eDNA content from mouse peritoneal lavage fluid with 1 μmol/L SYTOX Orange stain for 6 h was measured on a fluorescence plate reader. (**F–H**) Fluorescence microscopy images of ET production were visualized from mouse MФ (**F**), neutrophils (**G**) or a mixture of MФ and neutrophils (**H**) in the peritoneal lavage fluid *ex vivo* at 37 °C for 4 h, and they were visualized by fluorescence microscopy with a 20 × objective lens. ET formation was visualized in red (SYTOX Orange) and mouse peritoneal cells were visualized in blue with the Hoechst 33342 dye. (**I**) Fluorescence microscopy images of total peritoneal cells interacting with *S. aureus ex vivo* at 37 °C for 3 h were visualized by fluorescence microscopy with a 20 × objective lens. ET formation was visualized in red (SYTOX Orange), *S. aureus* Xen 29 with a GFP–producing plasmid was visualized in green and mouse peritoneal MФ was visualized in blue with the Hoechst 33342 dye. (**J–L**) The eDNA content was quantified from mouse MФ (**J**), neutrophils (**K**) or a mixture of MФ and neutrophils (**L**) in the peritoneal lavage fluid *ex vivo*. (**M**) The eDNA content was quantified from total peritoneal cells interacting with *S. aureus* and FOM *ex vivo*. The data are presented as the means ± standard errors derived from three independent experiments with One-Way ANOVA tests. **p < 0.01 compared between interest groups. *p < 0.05 compared between interest group.

*In vivo* FOM-mediated extra- and intracellular *S. aureus* killing studies were performed in a mouse peritonitis model. Mice were inoculated intraperitoneally (i.p.) with *S. aureus* Xen29 and then treated subcutaneously (s.c.) with FOM. The total, extra- and intracellular bacteria counts in the peritoneal lavage fluid were estimated. After a 4 h treatment with FOM, the total, extra- and intracellular bacteria counts were markedly reduced between the treated and untreated mice (*p* < 0.01, Fig. 5A–C). This result demonstrated that the scavenging effect of phagocytes against *S. aureus* was increased by FOM.

At the same time, ROS production and eDNA content in peritoneal lavage fluid were quantified. As shown in Fig. 5D, ROS production induced by *S. aureus* was significantly increased in a dose-dependent manner in the peritoneal lavage fluid following FOM treatment for 4 h (*p* < 0.01). Moreover, the eDNA content that was produced in the peritoneal lavage fluid following *S. aureus* infection was also significantly increased in a dose-dependent manner following FOM treatment (*p* < 0.01, Fig. 5E).

Next, we further corroborated ET production enhancement by FOM in peritoneal lavage fluid *ex vivo*. In uninfected mice, resident monocytes and MФ predominated in the peritoneal cavity; however, a few neutrophils were present. However, neutrophils can rapidly migrate and accumulate in the peritoneum in response to infection^5^. To observe ETs production, we separated MФ or neutrophils from the peritoneal lavage fluid. Fluorescence microscopy images showed that ET production from peritoneal lavage fluid MФ, neutrophils, or co-incubated MФ and neutrophils was enhanced by FOM, and this enhancement was decreased by AAPV (Fig. 5F–I). Moreover, phagocyte ET production was markedly increased with the addition of *S. aureus in vitro* (Fig. 5H,I). Surprisingly, ET production from the co-incubated MФ and neutrophils was markedly decreased compared with that of MФ or neutrophils alone (Fig. 5F–H). Further, the results from the eDNA content, using a fluorescence assay, were consistent with the fluorescence microscopy images (Fig. 5J–M). This study demonstrated that FOM strengthened ET production from the peritoneal lavage fluid of *S. aureus*-infected mice *ex vivo*. Together, these data suggested that FOM enhances extra- and intracellular killing of bacteria in a mouse peritonitis model *in vivo* and that it might be mediated by ET or ROS production, which was correlated with the *in vitro* study results.

### Real-time monitoring of the *in vivo* bactericidal effects of FOM

**Figure 6:**
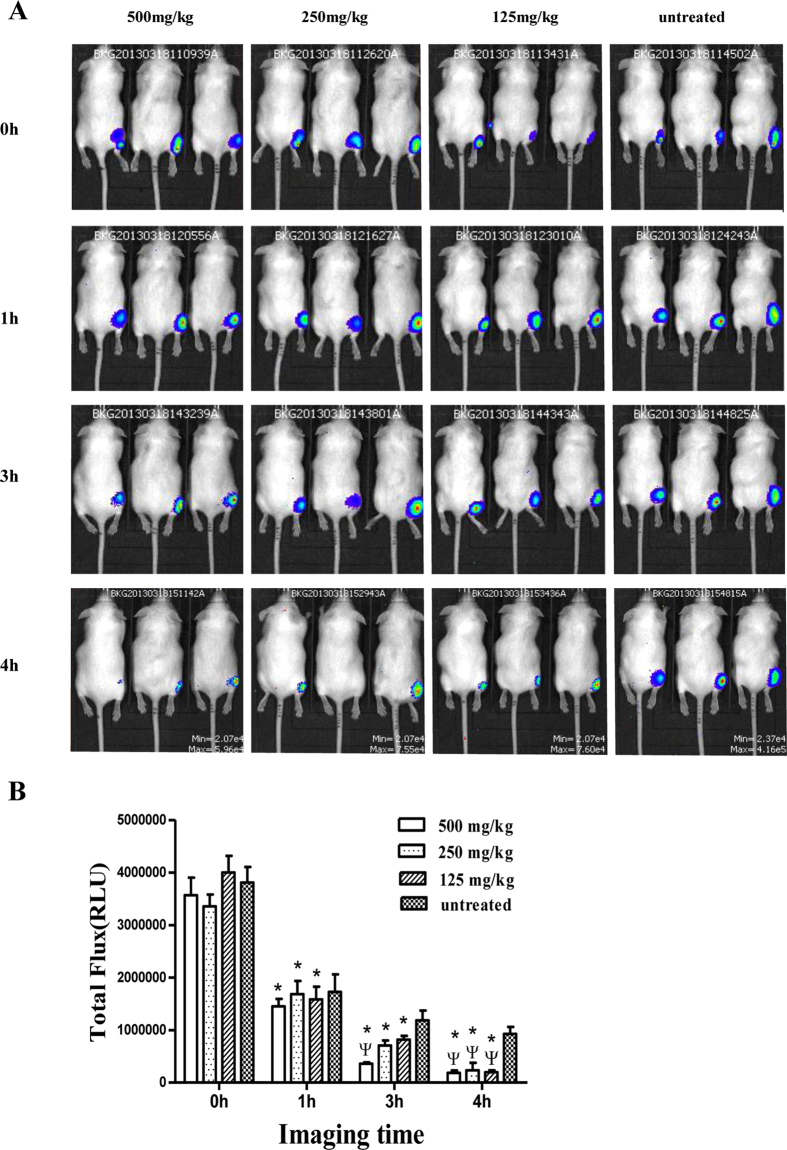
An *in vivo* bactericidal effect by FOM treatment was detected against *S. aureus*. (**A**) Real-time luminescence monitoring of *S. aureus* Xen29-infected mice that were treated with or without FOM. Bioluminescence generated by *S. aureus* Xen29 infection was detected in live animals using the IVIS imaging system at different time-points. Mice (n = 3 per group) were infected with *S. aureus* Xen29 in the thighs, and FOM at 0 mg/kg (untreated), 125 mg/kg, 250 mg/kg or 500 mg/kg was administered in 3 doses at 0.5 h post-infection. The mouse thighs were imaged at 0, 1, 3 and 4 h post-infection. (**B**) Dose dependency of times and RLU reductions with FOM treatment are shown. Mice (n = 3 per group) were infected with *S. aureus* Xen29 in the thighs, and FOM at 0 mg/kg (untreated), 125 mg/kg, 250 mg/kg or 500 mg/kg was administered in 3 doses 0.5 h post-infection 0.5 h. The RLU values were imaged at 0, 1, 3 and 4 h post-infection. The data are presented as the means ± standard errors derived from three independent experiments with One-Way ANOVAs. Treatment groups that showed statistically significant (*p* < 0.05) reductions in comparison with the 0-hour control group (*) and the same-hour control group (Ψ) are indicated.

To intuitively evaluate the bactericidal effects of FOM on *S. aureus* infections *in vivo*, real-time monitoring of mice using an IVIS imaging system was conducted. The image in Fig. 6A shows that luminescence was significantly reduced in *S. aureus* Xen29-infected mice that were administered FOM (125 mg/kg, 250 mg/kg or 500 mg/kg) 4 h post-infection. The result in Fig. 6B show that the RLU values, which were imaged at 1, 3 and 4 h post-infection, were significantly weaker compared with those at 0 h post-infection (*p* < 0.05). Additionally, the RLU values that were imaged at 3 h post infection with 500 mg/kg FOM treatment were significantly weaker than those of the same-hour control group (*p* < 0.05). Moreover, the RLU values that were imaged at 4 h post-infection with FOM (125 mg/kg, 250 mg/kg or 500 mg/kg) treatment were significantly weaker than those of the same-hour control group (*p* < 0.05). These results showed that FOM had a strong bactericidal effect against *S. aureus in vivo*.

## Discussion

It is well known that the combination of immune defences and antibiotics can contribute to the clearance of bacterial infections. Additionally, phagocytes and antibacterial agents may act synergistically in fighting infections, and antibacterial agents make bacteria more susceptible to clearance by phagocytes, even at sub-inhibitory concentrations^31^. Although several mathematical models examined the collective contribution of antibiotics and the immune response to the treatment of acute, self-limiting bacterial infections^32^, there have been only a few experimental studies that evaluated the mechanistic interactions between antibiotics and immune responses^33^,^34^,^35^. Thus, it is important to explore the immunomodulatory potential of antibacterial agents on phagocytes^36^.

Bacterial biofilms supply bacteria with significant resistance to host defences and antimicrobial agents^37^. In our study, the MBIC values of FOM against the tested strains were 2 to 16 fold higher than their relative MIC values, and the MBBC values of FOM against the tested strains were over 4 to 32 fold higher than their MBC values (Table 1). These results demonstrated that the susceptibility of FOM against *S. aureus* RBF cells was relatively lower compared with PLK cells. Additionally, when the bacteria to phagocyte (E:T) ratios were greater than 1:5, the RBF cells showed a strong anti-phagocytic ability compared with the PLK cells (*p* < 0.05, Table 2). These data indicated that *S. aureus* RBF cells displayed reduced susceptibility not only to certain antibacterial, as had been previously demonstrated^12^,^38^, but also to immune cells, most likely because of the thick extracellular polysaccharide matrix that facilitates adhesion to hydrophobic surfaces^39^. However, although the RBF cells lacked the overall structure of biofilms and lost most of their matrix, they also showed reduced susceptibility to immune cells compared with the PLK cells^40^.

FOM is a low-molecular-weight antibiotic with no reported toxicity and low binding to serum proteins^41^. Thus, it is both feasible and meaningful to investigate whether FOM can act as an antibiotic synergistic agent with phagocytes against bacterial infection. In this study, we found that FOM increased the bactericidal effect of MФ or human neutrophils cells against *S. aureus* RBF or PLK cells (Fig. 1). Other studies also showed that *Candida albicans* within biofilms are not only more resistant to phagocytic host defences but are also susceptible to the additive effects of phagocytes and an echinocandin^40^.

Physiological generation of ROS occurs either as by-products of (redox) reactions in various cell organelles, including mitochondria, peroxisomes, and endoplasmic reticulum, or by primary enzyme function, such as with oxidases and oxygenases. Plasma membrane-bound phagocyte NADPH oxidase was commonly thought to be the main source of ROS delivery into the extracellular space during respiratory bursts and into engulfed phagosomes for microbial killing^25^,^26^,^27^,^28^. The oxidative burst pumped electrons into the phagosome that was compensated by a flux of K ^+^ ions across the membrane in a pH dependent matter. This is an important trigger for the release of cationic granule proteins^42^. Anderson *et al*. showed that MФ exhibit similar characteristics with respect to *S. aureus*-induced ROS responses to human and mouse neutrophils^3^. In this study, we found that both *S. aureus* RBF and planktonic cells induced ROS in both MФ and neutrophils, and FOM enhanced the bacterial killing activity of phagocytes by increasing ROS production. This was similar to a previous report showing that FOM enhanced bactericidal ability by elevating extracellular reactive oxygen intermediate (ROI) production in neutrophils^43^.

Many bactericidal antimicrobials are known to share a common lethal pathway that involves the generation/accumulation of hydroxyl radicals, which cause oxidative damage to bacterial DNA^29^. In this experiment, we used HPF to determine whether FOM could generate hydroxyl radicals. HPF is a cell-permeable fluorescence probe that selectively detects highly reactive oxygen species (hROS), such as hydroxyl radicals^44^. The results showed that FOM induced hydroxyl radical production (Fig. 4A) in *S. aureus*-RBF or planktonic cells, and FOM could enhance the killing of *S. aureus* by hydroxyl radical production from *S. aureus* cells (Fig. 4B). As a bactericidal agent, the result of FOM-induced hydroxyl radical production is consistent with the conclusion that the generation of hydroxyl radicals is a common mechanism of bacterial cell death caused by bactericidal antibiotics^19^,^20^.

ET formation has recently been recognized as a novel defence mechanism in neutrophils^21^,^45^, MФ^46^, mast cells^47^ and eosinophils^48^. It was suggested that these structures are toxic to microbes and significantly contribute to the killing of several pathogens. It was demonstrated that ET formation is dependent upon NADPH oxidase activity^21^, and treatment with the NADPH oxidase inhibitor DPI could effectively inhibit NET production over longer incubation periods with *S. aureus* (by 30% at 2 h and 80% at 3 and 4 h). This evidence suggests that NET formation by *S. aureus* requires ROS in a time-dependent manner^49^. From our results, we observed that FOM promoted *S. aureus*-infected phagocytes to produce ETs in order to kill bacteria, and our results suggested that ET induction by *S. aureus* alone or with FOM for 3 h was dependent upon NADPH oxidase activity.

Several *in vitro* models using either human or animal cells have been developed to study the activity of antibiotics against intracellular *S. aureus*, and a corresponding *in vivo* model (murine peritonitis) has recently been described and tested with antibiotics, including linezolid, β-lactams, gentamicin, azithromycin, rifampicin, or dicloxacillin^5^,^6^,^50^. In this study, a detailed characterization of the extra and intracellular activities of FOM against *S. aureus* was carried out with a combination of *in vitro* (cultured MФ or neutrophils) and *in vivo* (mouse peritonitis) models.

It was demonstrated that phagocyte ETs can be formed *in vivo* and contribute to infection clearance^51^. In uninfected mice, resident monocytes and MФ predominate in the peritoneal cavity, with few neutrophils present. However, neutrophils can rapidly migrate and accumulate in the peritoneum in response to infections and chemical stimuli^52^. We evaluated whether FOM affected the ET production from the peritoneal lavage fluid of *S. aureus*-infection mice *ex vivo*. Fluorescence microscopy images and eDNA content quantitation showed that the ET production from MФ, neutrophils or MФ plus neutrophils were strengthened by FOM in the peritoneal lavage fluid (Fig. 5). MФ or neutrophil ET production *ex vivo* was consistent with the results obtained *in vivo*. However, we were surprised that the ET production by co-incubated phagocytes was decreased compared with that of MФ or neutrophils alone. Recent research showed that MФ were capable of NET clearance^53^, and *Newman et al.* showed that human senescent neutrophils were phagocytosed *in vitro* by human or rabbit MФ and were digested quickly in phagosomes^54^. It was also reported that, *in vitro*, the MФ uptake of intact, apoptosing neutrophils or of purified neutrophils granules resulted in the enhancement of MФ anti-*M. tuberculosis* activity^55^. Moreover, recent studies demonstrated that MФ could devour the microbicidal molecules of neutrophils to enhance their comparatively limited antimicrobial capacity, which has beneficial effects on the protective host immune response^56^. From our results, the ET decreases from the co-incubated phagocytes might be because the MФ cleared the neutrophils or the neutrophil ETs.

In conclusion, our findings indicate that FOM is an antibacterial synergistic agent of MФ that acts against *S. aureus* planktonic cells and biofilms. As a bactericidal agent, FOM itself induced hydroxyl radical production in *S. aureus*, and FOM stimulated *S. aureus*-infected phagocytes to produce more ROS and ETs in order to kill the bacteria. Moreover, the extra- and intracellular bactericidal activity and ROS and ET production were increased by FOM *in vivo.* Further, the FOM bactericidal activity was measured under pathological conditions, and real-time monitoring of mice corroborated that FOM was effective against *S. aureus* infection *in vivo*. Together, these findings underscore the importance of the additional effects of FOM on the bactericidal capacity of phagocytes, and simultaneously provided evidence for further studies regarding the interactions between antibiotics and the immune response.

## Methods

### Ethics statement

Mice were housed in micro-isolator cages and received food and water. The laboratory temperature was 24 ± 1 °C, and the relative humidity was 40–80%. All animal studies were conducted according to the experimental practices and standards that were approved by the Animal Welfare and Research Ethics Committee at Jilin University (no: IZ-2009-008). The protocols were reviewed and approved by the committee. All of the animal studies were performed under isofluorane anaesthesia except mouse peritonitis model experiments (diethyl ether was used), and every effort was made to minimize suffering.

### Strains and growth conditions

*S. aureus* SA113, Xen29, SA113 ∆*ica* (an isogenic *ica* deletion mutant) and RN6390 were used in this study. SA113, Xen29, and RN6390 that were transformed with a green fluorescent protein (GFP)–producing plasmid (pCN57) were used. Bacterial cells were grown at 37 °C in Tryptic Soy Broth (TSB) (Oxoid, Basingstoke, UK) or TSB with 0.25% glucose. FOM was purchased from Sigma-Aldrich and dissolved in sterilized water at a concentration of 40960 μg/ml under sterile condition, and it was stored at −20 °C until use.

### Microorganism preparation

Biofilms were established as previously described^57^. RBF cells were obtained as previously described^58^. For the metabolic assays, SA113, Xen29, RN63903 and SA113 ∆*ica* were used.

### Preparation of phagocytes

Mouse peritoneal MФ were isolated from female BALB/c mice with thioglycolate treatment at 4 weeks of age by lavage with phosphate-buffered saline, as previously described^59^. The THP-1 monocytic cell line was purchased from the cell bank of the Chinese Academy of Sciences (Shanghai, China). Neutrophils were isolated (> 95% pure) from the peripheral blood of normal individuals by centrifugation using Polymorphprep^TM^ (Axis-shield PoC AS, Oslo, Norway) as recommended by the manufacturer^60^. THP-1 cells were differentiated to a MФ phenotype with 10 ng/ml phorbol myristate acetate (PMA; Sigma-Aldrich) at 37 °C for 6 h. MФ viability was 95%, as determined by trypan blue staining.

### PLK and biofilm antimicrobial susceptibility testing

To determine FOM MICs, microbroth dilution assays were performed in line with CLSI (formerly NCCLS) guidelines. MBC was identified with agar plate assays. The minimum biofilm inhibition concentration (MBIC) and the minimum biofilm bactericidal concentration (MBBC) evaluations were performed as previously described^56^. The assays were repeated in triplicate.

### *S. aureus* incubation with FOM and/or phagocytes

*S. aureus* RBF and PLK cells were incubated with phagocytes at E:T ratios of 10:1, 1:1, 1:5, or 1:10 at 37 °C in a humidified, 5% CO_2_ incubator for 2 or 22 h. *S aureus* RBF or PLK cells were incubated with phagocytes at E:T ratios of 1:1, 1:5 and FOM (1/2 MIC, 1/4 MIC and 1/8 MIC) at 37 °C in a humidified, 5% CO_2_ incubator for 22 h.

### XTT metabolic assay

After incubation, phagocytes were lysed hypotonically, and the viability was assessed by a modification of the XTT (2,3-bis [2-methoxy-4-nitro-5-sulfophenyl] 2H-tetrazolium-5- carboxanilide; 0.25 mg/ml) metabolic assay, using the Q0 coenzyme (2,3-dimethoxy-5-methyl- 1,4-benzoquinone; 40 mg/ml) as the final electron acceptor agent. Optical densities were measured with a spectrophotometer (An-thos 2000; Labtech) at 450 nm.

### Fluorescence microscopy

The phagocytosis assay was visualized with fluorescence microscopy in Xen29-infected MФ or with 4 μg/ml FOM treatment for 2 h (6 h). The cells were stained with Hoechst 33342 (10 μg/ml) (Sigma-Aldrich) for 5 min at 37 °C. The images were collected with an Olympus BX53 fluorescence microscope (Olympus, Tokyo, Japan) with 20 or 40× objective lenses.

### ET release

Phagocytes were attached to coverslips, as described earlier, for the phagocytosis assay. Afterward, the cells were treated with 1 × 10^6^ GFP-expressing Xen29 cells alone or in combination with 4 μg/ml FOM treatment for 3 h. PMA (100 nM) was added to phagocytes as a positive control. Extracellular DNA (eDNA) was stained with 1 μmol/L SYTOX Orange (Life Technologies) for 20 min at 37 °C. Phagocytes were visualized in blue with Hoechst 33342 (5 μg/ml). ET release was analysed using an Olympus BX53 fluorescence microscope (Olympus, Tokyo, Japan) with a 20× objective lens. For eDNA content measurement, phagocytes were coincubated with *S. aureus* in white 24-well plates. Released DNA was stained with 1 μmol/L SYTOX Orange, and the fluorescence was measured in a fluorescence reader (Tecan Infinite F200) with emission and absorption filters of 540 and 575 nm, respectively.

### Scanning electron microscopy

Coverslips were coated with 10% poly-D-lysine. Neutrophils or MФ with *S. aureus* were prepared as above, but they were incubated on coverslips. The incubated cells were fixed with 2.5% glutaraldehyde at −4 °C for 30 min, fixed with 1% osmium tetroxide at −4 °C for 30 min, dehydrated with a graded ethanol series, critical-point dried, and covered with a gold film by sputter coating. The specimens were then analysed with a scanning electron microscope (Hitachi S-3400N, Japan).

### ROS detection in the phagocytes and hydroxyl radical formation in the *S. aureus* following FOM treatment

Intracellular ROS production from phagocytes or *S. aureus* hydroxyl radical production was detected with 2′, 7 dichlorofluorescein diacetate (DCFH-DA) (Sigma) or 3′-(p-hydroxyphenyl) fluorescein (HPF, Invitrogen, Eugene, OR, USA). Ten nanomolar DPI (the NADPH oxidase inhibitor) pretreated-phagocytes (1 × 10^5^ cells/well) were exposed to *S. aureus* or 4 μg/ml FOM for 3 h. PMA was utilized as a positive control. *S. aureus* were pretreated with 100 mM thiourea for 2 h, which is a hydroxyl radical scavenger. 10 μM HPF or 5 μM DCFH-DA was added to the cultures at 37 °C for 40 min. Hydroxyl radical or ROS formation was detected with a fluorescent reporter (Tecan infinite F200) at 490 nm excitation and 520 nm emission wavelengths.

### Phagocytosis and phagocyte bactericidal activity assays

The experimental phagocytosis and intra- and extracellular killing conditions included bacterium alone, as a control group, and cell groups that were treated with Xen29 or FOM (4 μg/ml)-treated Xen29. Then, each group was treated with 100 U/ml DNase I or 10 μg/ml cytochalasin D (Sigma) for 3 h at 37 °C. The experiments were repeated five times. For the gentamicin protection assay, phagocyte pellets were resuspended with PBS containing 50 μg/ml gentamicin to kill any adherent extracellular bacteria and washed twice to remove the gentamicin. All of the bacterial samples were serially diluted and plated onto TSB agar to determine the CFU counts. The percentage of phagocytosis or extracellular killing by the phagocytes in the wells containing DNase I to inhibit ET formation or cytochalasin D to inhibit phagocytosis was determined using the equation (1−CFU_extracellular_/CFU_control_) × 100. The percentage of intracellular killing by MФ or neutrophils in replicated wells containing DNase I was determined using the equation [1−CFU_intracellular_/(CFU_control_ − CFU_extracellular_)] × 100.

### Separation of intra- and extracellular *S. aureus* in peritoneal fluid following peritonitis induction in mice

The mouse peritonitis model, performed as described previously, was used for all *in vivo* infection studies^5^,^6^. In short, female BALB/c mice were inoculated 2 h before antibiotic treatment with an injection of a total of 1 × 10^8^ CFUs, intraperitoneally (i.p.), unless stated otherwise (with an injection volume of 0.5 ml). Antibiotic treatments were administered subcutaneously (s.c.). The mice were euthanized, and the peritoneal fluid was collected by injecting 2.0 ml PBS i.p. The number of total, extra- and intracellular CFUs in the peritoneal fluid was quantified as described previously^5^,^6^.

### ET production from the peritoneal lavage fluid of *S. aureus*-infection mice *ex vivo*

MФ, neutrophils or phagocytes from mouse peritoneal lavage fluid were cultivated in 24-well microplates, including coverslips, at 37 °C for 4 h *ex vivo*. Phagocytes from mouse peritoneal lavage fluid were cultivated with *S. aureus* cells at E:T ratios of 10:1 at 37 °C for 3 h *ex vivo*.

### *In vivo* imaging

An inoculum of ~1 × 10^8^ CFU *S. aureus* Xen29 cells (Xenogen Corporation, Alameda, CA, USA) per back was delivered subcutaneously (s.c) in 200 μl of sterile saline to each thigh. The s.c. treatment was initiated 1 h post-infection, followed by 500 mg/kg, 250 mg/kg, 125 mg/kg and 0 mg/kg of FOM at other thigh. At approximately 0, 1, 3 and 4 h after infection, the animals were anesthetized with 2% isofluorane, and bioluminescent images, which were generated by the Xen29 infection in the dorsal side of the infected thighs of the live animals, were recorded for five minutes using the IVIS imaging system (Xenogen Corporation). Very intense bioluminescence signals were displayed as red and low-intensity signals were displayed as blue. The total photon emission (relative light units, RLUs) was quantified from the defined regions of interest using the Living Image software (Xenogen Corporation). The assay was repeated in triplicate.

### Statistical analysis

Comparisons of the mean values from three experiments were statistically evaluated by analysis of variance, followed by One-Way ANOVA analysis or independent-sample T tests. Differences with 2-sided *P* values < 0.5 were considered statistically significant. All of the statistical analyses were performed with the SPSS software (version 11.5; SPSS).

## Additional Information

**How to cite this article**: Shen, F. *et al*. Fosfomycin enhances phagocyte-mediated killing of *Staphylococcus aureus* by extracellular traps and reactive oxygen species. *Sci. Rep.* **6**, 19262; doi: 10.1038/srep19262 (2016).

## Figures and Tables

**Table 1:** FOM activities against PLK cells, RBF cells and biofilms, as determined by microbroth dilution assays and the agar plate method.

Strain	PLK		Biofilm		RBF	
	MIC	MBC	MIBC	MBBC	MIC	MBC
RN6390	16	64	32	>1024	16	128
Xen29	16	256	128	>1024	16	512
SA113	16	64	256	>1024	32	256
SA113∆*ica*	16	32				

**Table 2:** Comparative effects of human THP-1 cells, neutrophils and mouse MФ on damage to *S. aureus* PLK and RBF cells, as determined by the XTT Assay.

Treatment time, E:T ratio	Human THP-1		Mouse MФ		Human neutrophil	
	PLK	RBF	PLK	RBF	PLK	RBF
2h						
10:1	89.4 ± 1.1	83.1 ± 0.8**	73.0 ± 1.5	64.9 ± 2.4**	90.5 ± 2.2	81.5 ± 2.1*
1:1	81.0 ± 1.3	69.5 ± 1.3**	54.8 ± 2.3	41.8 ± 2.3**	84.4 ± 2.0	71.4 ± 2.4**
1:5	66.7 ± 1.2	54.1 ± 1.4**	44.4 ± 1.2	31.3 ± 1.4**	76.0 ± 2.2	62.7 ± 1.7**
1:10	35.8 ± 1.6	26.2 ± 1.9**	17.9 ± 1.4	15.1 ± 2.0	63.8 ± 3.5	47.2 ± 3.7**
22h						
10:1	83.3 ± 0.7	63.1 ± 0.9**	67.7 ± 1.9	60.5 ± 1.1**	70.5 ± 3.6	51.5 ± 3.8**
1:1	74.1 ± 1.5	63.1 ± 0.8**	50.3 ± 2.4	33.6 ± 1.1**	51.1 ± 3.5	34.4 ± 2.0**
1:5	58.3 ± 0.5	51.3 ± 0.9**	39.3 ± 1.4	23.8 ± 1.4**	36.4 ± 3.6	26.0 ± 2.2*
1:10	15.0 ± 1.1	12.4 ± 1.4	15.0 ± 1.1	12.4 ± 1.4	19.5 ± 7.0	11.5 ± 2.2

NOTE. The results are expressed as the percentage of damage to *S. aureus* PLK cells and RBF cells induced by human THP-1 cells, neutrophils and mouse MФ compared with untreated controls (damage considered to be 0%) by analysis of variance with the One-Way ANOVA test. The data displayed as means ± standard errors of values from three experiments that were performed on different days. E:T ratio, effector to target cell ratio; XTT, 2,3-bis[2-methoxy-4-nitro-5-sulfophenyl] 2H-tetrazolium-5-carboxanilide.

**p < 0.01 significant difference between PLK cells and biofilm cells.

*p < 0.05 significant difference between PLK cells and biofilm cells.
